# Own and parental war experience as a risk factor for mental health problems among adolescents with an immigrant background: results from a cross sectional study in Oslo, Norway

**DOI:** 10.1186/1745-0179-2-30

**Published:** 2006-11-03

**Authors:** Lars Lien, Brit Oppedal, Ole Rikard Haavet, Edvard Hauff, Magne Thoresen, Espen Bjertness

**Affiliations:** 1 Institute of Psychiatry, University of Oslo, PO Box 1130, Blindern, 0318 Oslo, Norway; 2 The Norwegian Public Health Institute, Department of Mental Health, PO Box 4404, Nydalen, 0403 Oslo, Norway; 3 Institute of General Practice and Community Medicine, University of Oslo, PO Box 1130, Blindern, 0318 Oslo, Norway

## Abstract

**Background:**

An increasing proportion of immigrants to Western countries in the past decade are from war affected countries. The aim of this study was to estimate the prevalence of war experience among adolescents and their parents and to investigate possible differences in internalizing and externalizing mental health problems between adolescents exposed and unexposed to own and parental war experience.

**Method:**

The study is based on a cross-sectional population-based survey of all 10^th ^grade pupils in Oslo for two consecutive years. A total of 1,758 aadolescents were included, all with both parents born outside of Norway. Internalizing and externalizing mental health problems were measured by Hopkins Symptom Checklist-10 and subscales of the Strengths and Difficulties Questionnaire, respectively. Own and parental war experience is based on adolescent self-report.

**Results:**

The proportion of adolescents with own war experience was 14% with the highest prevalence in immigrants from Eastern Europe and Sub-Saharan Africa. The proportion of parental war experience was 33% with Sub-Saharan Africa being highest. Adolescents reporting own war experience had higher scores for both internalizing and externalizing mental health problems compared to immigrants without war experience, but only externalizing problems reached statistically significant differences. For parental war experience there was a statistically significant relationship between parental war experience and internalizing mental health problems. The association remained significant after adjustment for parental educational level and adolescents' own war experience.

**Conclusion:**

War exposure is highly prevalent among immigrants living in Oslo, Norway, both among adolescents themselves and their parents. Among immigrants to Norway, parental war experience appears to be stronger associated with mental health problems than adolescents own exposure to war experience.

## Background

During the last two decades, an increasing number of people immigrating to Europe and other Western countries have come from war thorn areas like Eastern Europe and Africa South of Sahara [1,2]. Many of the children and their families have been seriously traumatized by their pre-emigrating war experiences. A war exposes children to multiple traumatic events, like experiencing or witnessing violent acts or the results of violent acts as well as experiencing non-violent traumas like homelessness and starvation [3,4].

In the Bosnian war in 1994 almost 80% of the children surveyed experienced the death of a friend or family member and 73% were exposed to close shootings [4]. The same study found that witnessing killings, being in a threatening situation, knowing of raped or killed family members and being cold and having no food were related to self reported posttraumatic reactions. The different types of war exposure were related to different teacher and self reported adjustment reactions like aggressive behavior, depression and anxiety [4]. Hadi and Llabre found that during the Gulf war more than 80% of Kuwaiti children exposed to the violence of the Persian Gulf crises developed Posttraumatic Stress Disorder (PTSD) [5].

Long-term, follow-up studies of children acutely traumatized show that the psychopathology tends to increase acutely followed by a gradual reduction in symptoms [3]. These studies have also demonstrated that acute events that produce little changes in the social milieu tend to carry lower risk than either chronic ongoing traumatic events or other experiences that cause long-term disruptions in children's social environment [3]. A study of Cambodian refugees showed a dose-response relationship between trauma exposure and psychiatric disorders two decades after resettlement in the US [6].

It is not clear, however, to what extent psychological symptoms appearing during disasters interfere with children's daily life and functions and thus may be pathologic or alternatively, whether the symptoms may be considered to be "normal" reactions to abnormal events [3]. Allwood et al found in their study from Bosnia that children exposed to direct violence did not show more post-trauma reactions than children who experienced only nonviolent trauma [4]. Almedom and Summerfield argue that psychiatric labeling of children experiencing war is questionable, pathologizing and stigmatizing [7].

Oppedal et al reporting from Norway, were not able to detect any differences in mental health problems on group level among immigrant youth coming from conflict areas compared to other immigrant adolescents from labor sending countries [8]. In a review of mental health and adjustment problems of immigrant children the overall conclusion was that immigrant children do not seem to suffer from worse mental health than nonimmigrant children [9]. There is, however, subgroups of children who are at higher risk such as immigrant children whose families are in considerable conflict or turmoil or children arriving outside of a family context [9].

Parental mental health and susceptibility to parental mental distress are important predictors for the mental health of children [10]. In a literature review Perry and Ishnella [11] found that lower levels of PTSD in people experiencing disasters were associated with level of communities where individuals shared their experiences. The same applied to parental participation in the child's emotional recovery. The capacity to provide a consistent, predictable, and supporting environment was compromised if the family was disorganized and the child's primary caregiver was traumatized [11,12].

Studies of concentration camp survivors indicate that aspects of parental traumatic experiences might be transmitted to their children ("second generation syndrome") [13]. One of the mechanisms behind this "transmission" is lack of communication about the traumatic experiences within the family [14]. Children growing up in families where one of the parents has suffered a major trauma and there was lack of openness about the event, suffer more from pathologic identification with their traumatized parents than children where there was open communication about past events. Children from the former families tend to be less satisfied as kids, become more pessimistic as adults and suffer from more depressions than children from families with an open communication [14].

A study of adult immigrants to Oslo, Norway, found that one fourth of the study population was mentally distressed [15]. Past traumatic experiences was one risk factor associated with mental distress together with unemployment, recent negative life events, and economic problems [15]. We have surveyed immigrant adolescents from the same geographical area. Our hypothesis is that adolescents with war experience in their immigration history have more mental health problems than adolescents with no war experience and that adolescents growing up in families where one of the parents have war experiences also tend to have more mental health problems than adolescents growing up in families without war experiences.

The aim of this study among adolescents from different immigrant groups in Oslo, Norway was to:

• Estimate the prevalence of war experience among adolescents and their parents

• Investigate possible differences in internalizing and externalizing mental health problems between adolescents exposed and unexposed to own and parental war experience.

## Method

### Sample

The study was based on data from the youth part of the Oslo Health Study, a cross-sectional survey conducted by the Norwegian Institute of Public Health, the Municipality of Oslo, and the University of Oslo. All pupils in the 10^th ^grade in all schools of Oslo in 1999/2000 and 2000/2001 were included. As 10^th ^grade is compulsory in Norway, the present study included all 15–16 year olds in two cohorts. A total of 7,343 (88.3%) out of 8,316 eligible pupils participated in the study and answered the two four-page questionnaires during two school classes. 24% of the informants had immigrant background in the sense that they have two foreign born parents [2].

Because of an unfortunate error during the data-file preparation, information about gender was lost for 38 participants, and they were therefore excluded, leaving a total of 7,305 participants. For the purpose of this study we included all immigrant adolescents. Thus, the total study sample included 1,758 adolescents. Of these 722 (41%) are 2^nd ^generation in terms of being born in Norway of two foreign born parents. See Oppedal et al for further details on study design and methods [8].

### Measures

*Internalizing problems *were measured by the ten-item version of Hopkins Symptoms Check List (HSCL-10). The reliability was high (Cronbach α: .87) and the correlation with other instruments, including HSCL-90 has been found to range between 0.87 and 0.97 [16-18]. Students are asked if they during the last week have experienced for example to be "suddenly scared for no reason". Each item is rated on a scale from 1 (not at all) to 4 (extremely). A mean sum score for all 10 items of equal or above 1.85 has shown to be a valid predictor for mental distress among subjects aged 16–24 year of age. The cut-off level of 1.85 is corresponding to the 1.75 cut-off of the HSCL-25 [18,19].

*Externalizing problem *were measured by 10 items about hyperactivity and conduct problems from the Strength and Difficulties Questionnaire (SDQ). SDQ is a questionnaire for assessing mental health in children and adolescents. The reliability was α: .65. One of questions was as follows: I am restless; I cannot stay still for long (answer on the basis of what things have been like during the last 6 months). The rating scale for SDQ is from 1 to 3 with the options of not true, somewhat true and certainly true. We chose a cut-off point at the 90^th ^percentile of the study sample, as this has been applied in several other studies, including one Norwegian [20-22].

#### War experience, adolescents

The questions asked were as follows: Have you ever-experienced war or the consequences of war at first hand?

#### Parental war experience

Has one of your parents experienced war or the consequences of war at first hand? In addition to the possibilities to answer yes and no a third box called "don't know" was included.

In none of these questions there were any possibilities to add any further information.

#### Immigrant groups

The group of adolescents with both parents born outside of Norway represented 85 different nations, and were divided into six broad regional immigrant groups based on cultures and geographic origins: Western Countries, Eastern Europe, Middle East/North Africa, Sub Saharan Africa, the Indian Subcontinent and East Asia/Pacific. The Eastern European region includes a high proportion of immigrants from the Balkan. The dominating group in the Middle East/North Africa region are immigrants from Turkey and the same applies to Pakistani immigrants in the Indian Subcontinent regional group. In the East Asia/Pacific region, immigrants from Vietnam have a high share.

The average length of stay in Norway for the 1^st ^generation immigrants was 8.7 years (SD = 3.9). Immigrants from Sub Saharan Africa had the shortest length of stay with 7 (3.9) years, with Western countries and Eastern Europe coming next with 8.0 (3.8) and 8.4 (3.8) years, respectively. Longest average stay in Norway had 1^st ^generation immigrants from the Indian Subcontinent with 8.9 (3.9) years and East Asia/Pacific with 9.2 (3.4) years.

#### Gender

We have stratified on gender because we anticipate sex differences in mental distress and in exposure to war experiences.

#### Missing

For 44 (2.5%) of the adolescents there is missing information on internalizing and 123 (7.3%) on externalizing mental health problems. On the questions of war experience 176 (10%) are missing of adolescents' own experience and 159 (9%) of parental experience.

### Data analysis

Mean scores for internalizing and externalizing mental health problems across war experience were analysed with one-way ANOVA. Tukey post hoc test was performed on the differences in parental war experience. The level of significance was set to p ≤ 0.05, CI = 95%.

### Ethics

The study protocol was reviewed by the Regional Committee for Medical Research Ethics and approved by the Norwegian Data Inspectorate. The study has been conducted in full accordance with the World Medical Association Declaration of Helsinki.

## Results

### Prevalence of war experience

Boys and girls reported almost the same amount of both own and parental war experience (Table 1). Adolescents from Eastern Europe and Sub Saharan Africa reported the highest level of own war experience, while the Indian Subcontinent and East Asia/Pacific were lowest. Parental war experience was most prevalent in children from Sub Saharan Africa and South East Asia/Pacific and lowest among Western countries.

**Table 1:** Prevalence (CI) of war experience by adolescents own and parental experience across immigrant regions.

	Own war experience		Parental war experience			
	Yes**		Yes**		Don't know**	
Region*	*Boys (736)*	*Girls (780)*	*Boys (744)*	*Girls (787)*	*Boys (744)*	*Girls (787)*
*Western Countries n = 107*	12% (3–21)	6% (0–13)	21% (10–32)	12% (3–21)	14% (4–24)	12 (3–21)
*Eastern Europe n = 148*	35% (23–47)	37% (26–48)	43% (31–55)	46% (34–58)	21% (10–32)	22% (11–33)
*Middle East/North Africa n = 376*	17% (11–23)	20% (11–29)	33% (28–40)	37% (30–44)	32% (23–41)	25% (18–32)
*Sub Saharan Africa n = 168*	34% (22–46)	26% (16–36)	60% (48–72)	45% (33–57)	23% (11–35)	24% (13–35)
*Indian Sub-continent n = 720*	8% (5–11)	5% (3–7)	25% (20–30)	23% (18–28)	34% (28–40)	40% (33–47)
*South East Asia and Pacific n = 165*	6% (2–11)	1% (0–3)	57% (46–68)	45% (34–56)	22% (12–32)	29% (17–41)
**TOTAL**	**103** **14% (11–17)**	**102** **13% (10–16)**	**255** **34% (31–37)**	**251** **32% (30–34)**	**216** **29% (25–33)**	**241** **31% (27–35)**

*Adolescents from the Latin American countries were excluded because of few individuals in the group (n = 49). **The categories were yes and no for own, and yes, don't know and no for parental war experience.

Parental war experience was more prevalent than adolescents' own war experience. In addition to the 33% of adolescents reporting parental war experience there were 220 (28.2%) boys and 248 (30.3%) girls who stated that they did not know whether their parents had any first hand war experience.

More girls than boys were reporting internalizing mental health problems, while opposite pattern was found for externalizing mental health problems (Table 2). There were also statistically significant differences between the immigrant regions. For internalizing problems both boys and girls from South East Asia reported most and boys from the Indian Subcontinent and girls from Western countries least problems. For externalizing mental health problems there was only statistically significant regional differences in prevalence among girls. The highest prevalence was found in immigrant girls from Middle East/North Africa and the lowest for Sub Saharan Africa.

**Table 2:** Percent (and number) with internalizing and externalizing mental health problems.

	Internalizing		Externalizing	
Region	Boys (736)*	Girls (780)*	Boys (744)*	Girls (787)*
*Western Countries n = 107*	10.7% (6)	20.0% (10)	21.2% (11)	14.0% (7)
*Eastern Europe n = 148*	14.3% (10)	20.5% (16)	12.7% (8)	10.3% (7)
*Middle East/North Africa n = 376*	10.9% (19)	33.0% (64)	11.1% (18)	14.5% (27)
*Sub Saharan Africa n = 168*	14.3% (11)	25.9% (22)	10.1% (7)	5.1% (4)
*Indian Sub-continent n = 720*	8.8% (31)	25.2% (86)	15.5% (53)	6.7% (22)
*South East Asia and Pacific n = 165*	23.1% (18)	40.2% (35)	14.3% (11)	8.2% (7)
**TOTAL**	**11.8% (95)**	**27.9% (233)**	**14.1% (108)**	**9.3% (74)**

* The difference between the regions are statistically significant at p < 0.05.

### War experience and mental health problems

**Figure 1:**
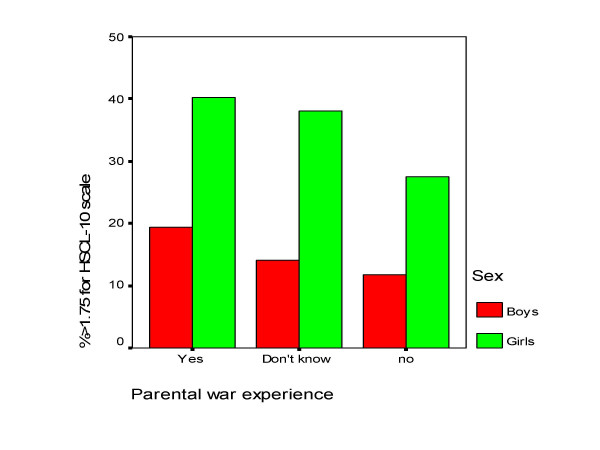
Percent with internalizing mental health problems across parental war experience and sex.

**Figure 2:**
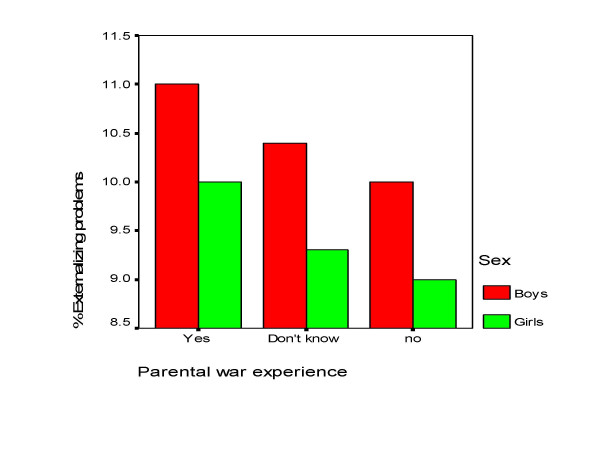
Percent with externalizing mental health problems across parental war experience and sex.

Adolescents with war experience as compared with non-exposed had higher mean scores for both internalizing and externalizing mental health problems among both boys and girls, but the only statistically significant difference was found for externalizing problems in boys (Table 3). For parental war experience there was a linear relationship in both internalizing and externalizing mental health problems through the three response categories "no", "don't know", and "yes" (Figure 1 and Figure 2). The differences between the groups were statistically significant for internalizing problems in both boys and girls and for externalizing problems in boys only. To test whether the effect of parental war experience was due to own war experience or differences in socioeconomic status we controlled for own war experience and parental educational level. The association between internalizing problems and parental war experience, however, remained statistically significant after controlling for these two factors (data not shown).

**Table 3:** Crude mean scores, with lower and upper 95% Confidence Interval (CI) of internalizing and externalizing mental health problems across adolescent's own and parental war experience and where both the adolescent and parents had war experience.

		Mental health problems							
		Internalizing problem				Externalizing problems			
Own war experience		*n*	*Mean*	*Lower CI*	*Upper CI*	*N*	*Mean*	*Lower CI*	*Upper CI*
*Boys*	*Yes*	106	1.43	1.33	1.53	108	6.60*	5.96	7.25
	*No*	652	1.34	1.31	1.38	655	5.78	5.52	6.02
*Girls*	*Yes*	103	1.66	1.55	1.77	105	5.76	5.22	6.30
	*No*	689	1.63	1.59	1.68	701	5.48	5.26	5.70
Parental war experience***									
*Boys*	*Yes*	274	1.43**	1.38	1.49	277	6.30*	5.91	6.69
	*Don't know*	214	1.33	1.27	1.39	215	5.79	5.33	6.24
	*No*	279	1.29	1.25	1.34	280	5.52	5.15	5.89
*Girls*	*Yes*	263	1.73**	1.65	1.81	265	5.56	5.19	5.92
	*Don't know*	236	1.66	1.58	1.73	246	5.72	5.35	6.09
	*No*	299	1.55	1.49	1.60	302	5.32	4.99	5.65
**Own and parental war experience**									
*Boys*	*Yes*	92	1.44	1.33	1.55	93	6.51	5.82	7.19
	*No*	271	1.30	1.24	1.35	271	6.49	5.84	7.18
*Girls*	*Yes*	93	1.70	1.58	1.82	95	5.52	4.96	6.07
	*No*	295	1.55	1.49	1.62	298	5.30	4.96	5.64

F-test: * p < 0.05 and ** p = 0.001. *** Post-hoc test (Tukey) showed that the differences among the parental group were between those with and without parental war experience.

The difference between the groups with parental war experience was significant between those with and without parental war experience, tested with Tukey post hoc test.

In separate analyzes we compared the group with both own and parental war experience with the group who reported neither own or parental war experience. The difference for this group was close to the difference between the adolescents exposed and unexposed for parental war experience. The difference was, however, not statistically significant.

## Discussion

This study shows that war experience among adolescents and their parents are prevalent in the Norwegian immigrant population and at the same level for boys and girls. It also shows that war experience might be associated with mental health problems in 10^th ^grade adolescents. Although pupils with war experience had higher mental health problem scores than the pupils not exposed to war, it was parental war experience that had the most profound effect, especially on internalizing mental health problems. Boys are different from girls with respect to externalizing, but not internalizing mental health problems, both for own and parental war experience.

The number of children affected by war in combat areas is high [3]. Most studies have analyzed prevalence data on war experience in the war-affected countries. These figures are therefore difficult to compare with our study of adolescents emigrating from conflict areas. Allwood et al for example, found that 73% of children living in Sarajevo, Bosnia during the 1994 siege were exposed to close shootings [4]. In our study 35% of the adolescents from Eastern Europe reported war experience. The difference is probably due to the fact that many of the adolescents coming to Norway did not live in war-affected areas.

Parental war experience is also difficult to compare with other studies, but we do have Norwegian studies of war experience among the adult population of immigrants. These figures show that 34% of the males and 18% of the females (25% for males and females together) in the adult population reported a traumatic history of war and/or torture [15]. This is different from our results where 33% reported that one of their parents had first hand war experience and might be due real differences in the two populations or that the adolescents perception of war, which is important in this study, are different from the real figures.

The finding that only parental war experience was significantly associated with internalizing mental health problems are in line with other research on the effects of war. Several studies have shown that children and adolescents adopt well after war trauma and have greater resilience than adults [3,12,23,24]. These studies also point to the conclusion that more than war experiences, these children suffer from possible post war deprivation, being in camps with shortage of food, electricity and water [24].

In the present study we lack the pre-immigration history and are therefore not able to confirm whether the adolescents have suffered from material deprivation before immigrating to Norway. What we know is that major life events like war experiences might have disruptive affects on families and that this might affect children and adolescents negatively [11,12,25]. Dysfunctional family interaction might be mediated via lack of openness and communication about the traumatic experiences within the family [14].

The high numbers of adolescents that have answered don't know to the question on parental war experience might indicate some lack of openness about traumatic events in the immigrant families. This possible lack of openness does not, however, result in more mental health problems among those answering don't know compared to the adolescents that know about that their parental war experience, rather the opposite. Other mechanism might therefore be more important in mediating the association between parental war experience and mental health problems than lack of openness.

After world war II researchers became interested in the second generation syndrome, whereby children of surveyors from Holocaust camps developed more mental health problems than their peers [13,26,27]. These studies were done on people returning back to their home country after been traumatized. In our case, many of the parents with war experience emigrate as a result of the war fair taking place in their home country. Coming to Norway, other problems arise like adapting to a new culture, getting a paid job etc [8,15].

The strength of this study was the high response rate (88.3%) from all 10^th ^grade students in Oslo for two consecutive years. Selection problems are thus a minor problem in the present study and it was not likely that the observed differences were due to selection bias. Furthermore, two well-validated questionnaires were applied, the HSCL-10 and SDQ, to assess mental health problems which was the main outcome variable of the study. A specific problem with cross-sectional studies is that those who report exposure also report outcome at the same point in time, thereby limiting the possibility to decide causation. In this study, however, the exposure lays many years back and it might therefore be more difficult to interpret the association between mental health problems and war involvement, especially when we lack information on the association closer to the war event.

Due to the time lag between exposure and outcome recall bias might be a problem. War experience is, however, an extremely extraordinary event most people will remember. It might be more difficult of course, to recognize parental war experience. There might also be some cases of misclassification due to the need to make up a war history for the purpose of being eligible for asylum. Another problem is that we lack information about timing, duration and intensity of exposure. Children or parents experiencing devastating civil war are classified with the same exposure as a father taking part in peace keeping missions. A third problem is that our response variables, SDQ and HSCL-10 might not be good enough to capture the effect of children's own and their parents' war experiences.

With a different cultural background and Norwegian, not as mother tongue, it might be difficult for the non-Norwegian groups to fully understand and comprehend the meaning of especially the HSCL-10 and SDQ parts of the questionnaire. Many of the questions might be culturally sensitive and the questionnaire has not been validated in a non-Norwegian setting. Earlier studies indicate that immigrants have a response style similar to that of the population of their host country. When interviewed in their own ethno-cultural setting, however, the response style is more towards the style of their country of origin [8].

Taken together, the limitations considered will most likely attenuate the results making the association between war experiences and mental health problems weaker.

## Abbreviations

HSCL 10 = Hopkins Symptoms Check List

SDQ = Strength and Difficulties Questionnaire

## Declaration of competing interests

The author(s) declare that they have no competing interests.

## Authors' contributions

LL carried out the statistical analyses and drafted the manuscript. BO and MT took part in the statistical analyses and commented on the drafts. ORH and EH took part in writing the methods section and commented on the drafts. EB took part in planning the study, participated in its design and coordination, and commented on the drafts. All authors read and approved the final manuscript.

## References

[B1] Carballo M Divino JJ Zeric D Migration and health in the European Union Tropical Medicine & International Health 1998 3 936 44 10.1046/j.1365-3156.1998.00337.x 9892278

[B2] Lie B Immigration and immigrants 2002 (Innvandring og innvandrere, 2002) 2002 Statistic Norway

[B3] Caffo E Belaise C Psychological aspects of traumatic injury in children and adolescents Child & Adolescent Psychiatric Clinics of North America 2003 12 493 535 10.1016/S1056-4993(03)00004-X 12910820

[B4] Allwood MA Bell-Dolan D Husain SA Children's trauma and adjustment reactions to violent and nonviolent war experiences Journal of the American Academy of Child & Adolescent Psychiatry 2002 41 450 7 10.1097/00004583-200204000-00018 11931602

[B5] Hadi F Llabre M The Gulf crisis experience of Kuwaiti children: psychological and cognitive factors Journal of Traumatic Stress 1998 11 45 56 10.1023/A:1024453015176 9479675

[B6] Marshall GN Schell TL Elliott MN Berthold SM Chun CA Mental health of Cambodian refugees 2 decades after resettlement in the United States JAMA 2005 294 571 9 10.1001/jama.294.5.571 16077051

[B7] Almedom AM Summerfield D Mental well-being in settings of 'complex emergency': an overview Journal of Biosocial Science 2004 36 381 8 10.1017/S0021932004006832 15293381

[B8] Oppedal B Roysamb E Heyerdahl S Ethnic group, acculturation, and psychiatric problems in young immigrants Journal of Child Psychology and Psychiatry and Allied Disciplines 2005 46 646 60 10.1111/j.1469-7610.2004.00381.x 15877769

[B9] Guarnaccia PJ Lopez S The mental health and adjustment of immigrant and refugee children Child & Adolescent Psychiatric Clinics of North America 1998 7 537 53 9894054

[B10] Bijl RV Cuijpers P Smit F Psychiatric disorders in adult children of parents with a history of psychopathology Social Psychiatry & Psychiatric Epidemiology 2002 37 7 12 10.1007/s127-002-8208-8 11924749

[B11] Perry BD Azad I Posttraumatic stress disorders in children and adolescents Current Opinion in Pediatrics 1999 11 310 6 10.1097/00008480-199908000-00008 10439203

[B12] Meier E Effects of trauma and war on children Pediatric Nursing 2002 28 626 9 12593349

[B13] Major EF The impact of the Holocaust on the second generation: Norwegian Jewish Holocaust survivors and their children Journal of Traumatic Stress 1996 9 441 54 10.1007/BF02103657 8827648

[B14] Major EF [When a trauma becomes a taboo – is it pathogenetic? Communication in the family about psychological traumas]. [Norwegian] Tidsskr Nor Laegeforen 1995 115 56 7 7846663

[B15] Thapa S Hauff E Gender differences in factors associated with psychological distress among immigrants from low- and middle-income countries. Findings from the Oslo Health Study Social Psychiatry Psychiatric Epidemiology 2005 40 78 84 10.1007/s00127-005-0855-8 15624079

[B16] Lipman RS Covi L Shapiro AK The Hopkins Symptom Checklist (HSCL) – factors derived from the HSCL-90 Journal of Affective Disorders 1979 1 9 24 10.1016/0165-0327(79)90021-1 162184

[B17] Sandanger I Moum T Ingebrigtsen G Sorensen T Dalgard OS Bruusgaard D The meaning and significance of caseness: the Hopkins Symptom Checklist-25 and the Composite International Diagnostic Interview. II Social Psychiatry & Psychiatric Epidemiology 1999 34 53 59 10.1007/s001270050112 10073122

[B18] Strand BH Dalgard OS Tambs K Rognerud M Measuring the mental health status of the Norwegian population: a comparison of the instruments SCL-25, SCL-10, SCL-5 and MHI-5 (SF-36) Nordic Journal of Psychiatry 2003 57 113 118 10.1080/08039480310000932 12745773

[B19] Tambs K Moderate effects of hearing loss on mental health and subjective well-being: results from the Nord-Trondelag Hearing Loss Study Psychosomatic Medicine 2004 66 776 782 10.1097/01.psy.0000133328.03596.fb 15385706

[B20] Goodman R Gledhill J Ford T Child psychiatric disorder and relative age within school year: cross sectional survey of large population sample BMJ 2003 327 472 475 12946967 10.1136/bmj.327.7413.472 PMC188428

[B21] Goodman R Ford T Simmons H Gatward R Meltzer H Using the Strengths and Difficulties Questionnaire (SDQ) to screen for child psychiatric disorders in a community sample British Journal of Psychiatry 2000 177 534 539 10.1192/bjp.177.6.534 11102329

[B22] Ronning J Handegaard B Sourander A Morch W-T The Strengths and Difficulties Self-Repoted Questionnaire as a screening instrument in Norwegian community samples European Child & Adolescent Psychiatry 2004 13 73 82 10.1007/s00787-004-0356-4 15103532

[B23] Porter M Haslam N Predisplacement and postdisplacement factors associated with mental health of refugees and internally displaced persons: a meta-analysis JAMA 2005 294 602 612 10.1001/jama.294.5.602 16077055

[B24] Carballo M Smajkic A Zeric D Dzidowska M Gebre-Medhin J Van HJ Mental health and coping in a war situation: the case of Bosnia and Herzegovina Journal of Biosocial Science 2004 36 463 77 10.1017/S0021932004006753 15293387

[B25] Gilman SE Kawachi I Fitzmaurice GM Buka L Socio-economic status, family disruption and residential stability in childhood: relation to onset, recurrence and remission of major depression Psychological Medicine 2003 33 1341 55 10.1017/S0033291703008377 14672243

[B26] Weisaeth L The European history of psychotraumatology Journal of Traumatic Stress 2002 15 443 52 10.1023/A:1020909620364 12482182

[B27] Summerfield D War and mental health: a brief overview BMJ 2000 321 232 5 10903662 10.1136/bmj.321.7255.232 PMC1118225

